# Durability associated efficacy of long-lasting insecticidal nets after five years of household use

**DOI:** 10.1186/1756-3305-4-156

**Published:** 2011-08-05

**Authors:** Eliningaya J Kweka, Yousif E Himeidan, Aneth M Mahande, Beda J Mwang'onde, Shandala Msangi, Michael J Mahande, Humphrey D Mazigo, Mramba Nyindo

**Affiliations:** 1 Tropical Pesticides Research Institute, Division of Livestock and Human Diseases Vector Control, Mosquito Section, P.O. Box 3024, Arusha, Tanzania; 2 Kilimanjaro Christian Medical College, Tumaini University, P.O.Box 2224, Moshi Tanzania; 3 Department of Medical Parasitology and Entomology, Weill-Bugando University College of Health Sciences, P.O. Box 1464, Mwanza, Tanzania; 4 Entomology Unit, Faculty of Agriculture and Natural Resources, University of Kassala, P.O. Box 71, New Halfa, Sudan

## Abstract

**Background:**

Long-lasting insecticidal nets (LLINs) have been strongly advocated for use to prevent malaria in sub-Saharan Africa and have significantly reduced human-vector contact. PermaNet^® ^2.0 is among the five LLINs brands which have been given full approval by the WHO Pesticide Evaluation Scheme (WHOPES). The LLINs are expected to protect the malaria endemic communities, but a number of factors within the community can affect their durability and efficacy. This study evaluated the durability, efficacy and retention of PermaNet^® ^2.0 after five years of use in a Tanzanian community.

**Method:**

Two to three day- old non blood-fed female mosquitoes from an insectary susceptible colony (*An. gambiae *s.s, this colony was established at TPRI from Kisumu, Kenya in 1992) and wild mosquito populations (*An. arabiensis *and *Culex quinquefasciatus*) were used in cone bioassay tests to assess the efficacy of mosquito nets.

**Findings:**

The knockdown effect was recorded after three minutes of exposure, and mortality was recorded after 24 hours post-exposure. Mortality of *An. gambiae *s.s from insectary colony was 100% while *An. arabiensis *and *Cx.quinquefasciatus *wild populations had reduced mortality. Insecticide content of the new (the bed net of the same brand but never used before) and used PermaNet^® ^2.0 was determined using High Performance Liquid Chromatography (HPLC).

**Conclusion:**

The results of this study suggest that, in order to achieve maximum protection against malaria, public health education focusing on bed net use and maintenance should be incorporated into the mass distribution of nets in communities.

## Findings

Malaria vector-human contact reduction has been shown to have a significant impact in decreasing malaria transmission and disease prevalence [1-4]. However, the use of malaria intervention tools in Africa is affected by culture and the socioeconomic status of malaria endemic communities and these have influenced bed net ownership and utilization [5,6]. At present, malaria endemic countries in Africa are reporting a wider coverage of LLINs with some countries reporting coverage of more than 60% [4,6,7]. The wide coverage is attributed by the willingness of African government and donors to fund the scale up of LLINs distribution.

Investment has primarily been for long lasting technology bed nets, including PermaNet^® ^2.0 which has been evaluated in different setting in malaria endemic regions globally [7-11]. Today, WHOPES has given a full approval to PermaNet^® ^2.0 to be categorized as long lasting net. The PermaNet^® ^2.0 nets are made with polyester and coated with deltamethrin, whereas the Olyset^® ^nets, which are impregnated with permethrin, are made from polyethylene [12,13]. The other generations of LLITNs, the Netprotect^® ^(Icon-Life) are impregnated with deltamethrin and are made from polyethylene filaments, while the Interceptor^® ^nets are coated with alphacypermethrin and are made from polyester filaments [12,13]. Lastly, the Duranet^® ^made from polyethylene filaments and are coated with alpha-cypermethrin [13]. Many others are still in the developmental stages. Currently, some of the greatest challenges for the use of LLINs in different malaria endemic settings, particularly in Africa are net durability, retention and efficacy. In Tanzania, PermaNet^® ^2.0 (Vestergaard Frandsen Company, Thailand) is among the brands of LLINs distributed in the community and its durability, retention and efficacy status has not yet been evaluated at community level. Its efficacy was reported only immediately after distribution [12]. Long term data on its durability, retention and efficacy is required for improving the effectiveness of LLINs at the field level and this was the main objective of the present study.

**Figure 1:**
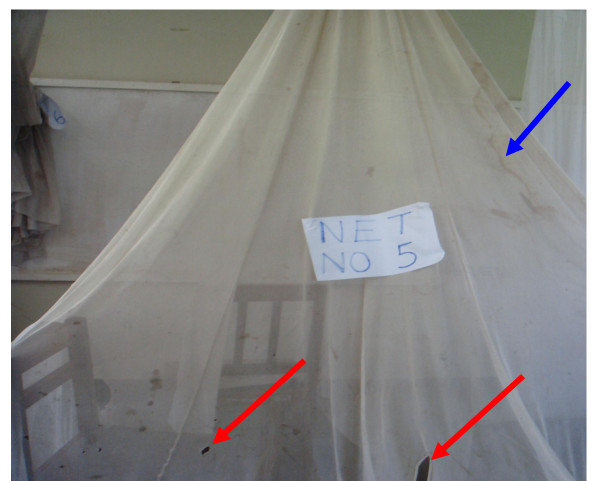
**A sample PermaNet^® ^2.0 collected from the community houses after 5 years of use**. A RED arrow points the holes which are due to mechanical contacts with bed angles while a BLUE arrow shows a mark on the net due to the firewood smoke indoors.

A total of sixty PermaNet^® ^2.0 were distributed in lower Moshi, north-eastern Tanzania to the households in 2005 and evaluated in 2010. The active ingredient measured among these nets at the time of distribution was 55 mg/m^2 ^of residual deltamethrin [12]. Of all (n = 60) the LLITNs (PermaNet^® ^2.0) distributed in the community, only 7 (11.7%) were still in use during the five years follow-up period. This result indicates a very poor retention of mosquito nets in Tanzanian community, a finding that is consistent with previous observations from several countries in Africa [14-16]. The durability was defined by the number of holes in nets used by the community for a period of five years. The seven retained nets were found to have a limited number of holes along bed angles contacts, meaning none had very good durability. Considering the strength of the high technology used to make the fibers of these nets [17], these holes could be attributed to the rough use of the community as it were found to be on the lower area of the net indicating tough mechanical tearing of the beds edges (Figure 1). It is known that, the presence of holes in long-lasting nets reduces the efficacy of protection against mosquitoes [18,19] despite high coverage in different parts of malaria endemic regions [20].

The New PermaNet^® ^2.0 induced high mortality for insectary- reared susceptible mosquito of *An. gambiae s.s *(100%). However, among the species tested, a wild population of *An. arabiensis *showed lower mortality rates than others. This is likely due to the pyrethroid resistance conferred by the 1014F-kdr mutation known to be predominant among mosquitoes population in this study area [21,22]. After use for a period of five years, PermaNet^® ^2.0 induced reduced mortalities in comparison to the new net. This was true for all strains of the three mosquito species tested (Table 1). Similar efficacy reductions have been reported for other types of LLINs after the same period [7,19]. Fifty community members (100%) responded to the questionnaire pertaining to the washing and use of the nets.

**Table 1:** Percentage knockdown (KD) and mortality at 24 hours post-exposure of wild populations and laboratory strain of the three mosquito species tested

Permanent^® ^2.0	*An. arabiensis *wild population		*Cx. quinquefasciatus *wild population		*An. gambiae *s.s insectary population	
	**%KD (95%CI)**	**%Mortality(95%CI)**	**%KD(95%CI)**	**%Mortality(95%CI)**	**%KD(95%CI)**	**%Mortality(95%CI)**

**New net**	66^a^(52.9-79.1)	46^ac^(32.2-59.8)	91^a^(83.1-98.9)	81^a^(70.1-91.9)	100^a^(100-100)	100^a^(100-100)
**Old net 1**	60^a^(46.4-73.6)	58^bc^(44.3-71.7)	73^bc^(.60.7-85.3)	62^b^(48.5-75.4)	100^a^(100-100)	30^b^(21.2-39.8)
**Old net 2**	60^a^(46.4-73.6)	49^cd^(35.1-62.7)	62^cde^(48.5-75.4)	58^b^(44.3-71.7)	100^a^(100-100)	47^cd^(32.4-51.3)
**Old net 3**	58^a^(44.3-71.7)	71^b^(58.4-83.6)	75^df^(62.9-87)	49^b^(35.1-62.9)	100^a^(100-100)	33^b^(19.3-43.7)
**Old net 4**	54^a^(.40.2-67.8)	49^ade^(35.1-.62.9)	38^b^(24.5-51.4)	44^b^(30.2-57.8)	100^a^(100-100)	50^ade^(41.3-58.8)
**Old net 5**	87^b^(77.7-96.3)	75^bf^(.62.9-87.0)	60^bef^(56.4-73.6)	54^b^(40.2-67.8)	100^a^(100-100)	33^b^(19.7-45.2)
**Old net 6**	54^a^(40.2-67.8)	83^bf^(72.6-93.4)	66^ef^(52.9-79.1)	56^b^(42.2-69.8)	100^a^(100-100)	33^a^(23.1-39.4)
**Old net 7**	60^a^(46.4-73.6)	73^ef^(60.7-85.3)	49^be^(35.1-62.9)	44^b^(30.2-57.8	100^a^(100-100)	53^be^(41.8-61.9)

*Number in the same row sharing the same superscript letter have no significant differences from each other (*P *> 0.05)

**Figure 2:**
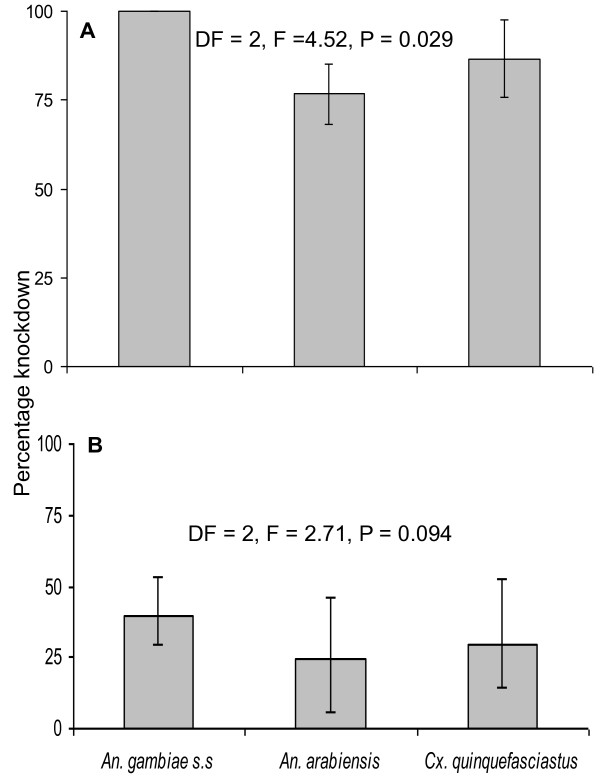
**Knockdown rate of laboratory unfed females mosquitoes of different mosquitoes species *An. gambiae *s.s *An. arabiensis *and *Culex quinquefasciatus *against (A)New and (B) five years used PermaNet^® ^2.0 at household level in Lower Moshi, north-eastern Tanzania**.

**Figure 3:**
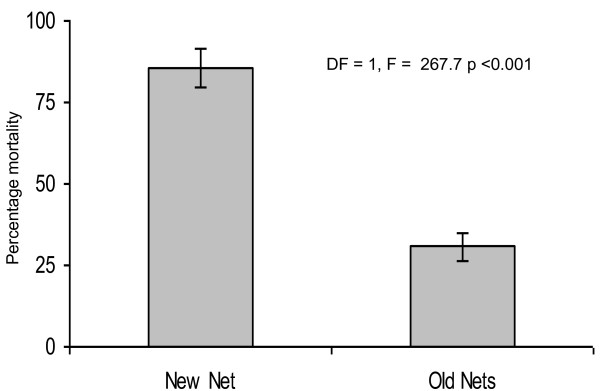
**Mortality at 24 hours post-exposure of unfed female of *Anopheles gambiae *s.s, *An. arabiensis, Culex quinquefasciatus *against New and five years used PermaNet^® ^2.0 at household level in Lower Moshi, north-eastern Tanzania**.

The knockdown rates effect varied among mosquito species for both the two types of nets assayed (Table 1) and in Figure 2. Mortality observed at 24 hours post-exposure for different populations and strains showed similar trends of knockdown effects for both new and used PermaNet^® ^2.0 (Figure 3). Regardless of species, a significant reduction was observed in the overall mortality when PermaNet^® ^2.0 used for five years compared with the new net of the same brand which was kept in store since the time of distribution (Figure 3).

The active ingredient found in PermaNet^® ^2.0 after use of five years was undetectable by HPLC procedure compared to 55 mg of active ingredient/m^2 ^of the new bed net of the same brand (Table 2). This could be attributed to use of strong detergents for washing net and the direct sun rays for drying them for a long period [7-9,17]. The firewood smoke and dust may also have contributed to the loss of insecticides in LLINs [7,8]. Generally, PermaNet^® ^2.0 has the potential to remain intact for a long period of time; and yet, washing techniques and other socioeconomic factors within the family or communities who use them may reduce their intended five years efficacy as per recommendation by WHOPES.

**Table 2:** Chemical ingredients of new and 5 years of field used net detected by HPLC technique

Net Condition	Number of washing	Deltermethrin content (mg/m^2)^	R-isomer	Bioefficacy Tests	
				KD_60_	FM 24 Hrs
**New Net**	0	66.5	3.6	84	100
**Old net A/01/F0**	130	n.d	< 0.5	14	20
**Old net A/02/F0**	130	n.d	< 0.5	10	8
**Old net A/04/F0**	130	n.d	< 0.5	0	10
**Old net A/05/F0**	128	n.d	< 0.5	0	10
**Old net A/07/F0**	130	n.d	< 0.5	0	10
**Old net A/09/F0**	126	n.d	< 0.5	0	10
**Old net A/10/F0**	130	n.d	< 0.5	0	10

n.d = No Deltermethrin detected, FM = mortality in 24 hours after contact bioassay.

### Bioassays procedures

On each net, four WHO contact bioassay cones were attached and a total of 10 mosquitoes were introduced into each cone. Two to three days old, unfed female of *An. gambiae *mosquitoes were used [23]. Wild populations of *An. arabiensis *and *Cx. quinquefasciatus *were collected from an area described by reduced susceptibility to pyrethroid insecticides [21,22]. The mosquitoes were exposed on each net for 3 minutes and then transferred to holding paper cups and provided with 10% sugar solution soaked in absorbent cotton wool [23-25]. Knockdown effects were recorded immediately after the 3 minutes and thereafter for 30 and 60 minutes post-exposure. Mortality was recorded after 24 hours post-exposure. Mosquitoes were considered knocked down or dead if they could not fly or could not stand upright on either the side or the bottom of the paper cups [23]. Untreated polystyrene net was used as a negative control for each bioassays test. Temperatures of 25°C ± 2 and relative humidity of 80% ± 10 were recorded during time of the tests using digital thermometer.

The mean percentage knock down (KD) at 3 minutes and mortality at 24 hours post-exposure were estimated for each of the evaluated bed net. One way analysis of variance (ANOVA) with Turkeys-Kramer HSD was used to compare the mortalities and KDs of mosquito species among the mosquito nets. All control mortalities were below 5%.

### Residual insecticide quantification

Four pieces of netting material were cut randomly from each net and then appropriately labeled with the name of the net, kept in individual envelops which were inserted into a single larger envelope and stored in the dark for subsequent residual insecticide quantification using high performance liquid chromatography (HPLC) [26,27]. For each test, the HPLC was performed using a piece of 5 × 5 cm from mosquito net with 0.15% grade water for determining deltamethrin iso-octan plus 1, 4 dioxan and dibutyl phthalate as internal standard [27]. Extracted samples were thoroughly shaken for uniform mixture and filtered through a 0.45 μm membrane filter suction pump. The filtered solution was aliquoted into 1 μL and then injected onto a normal phase isocratic HPLC machine. The insecticide quantification was achieved using an internal calibration curve based on UV detection.

## Conclusion

The results of this study suggest that, in order to achieve maximum protection against malaria, public health education focusing on bed net use and maintenance should be incorporated into the mass distribution of nets in communities. This may be effective for improving durability, washing, drying and retention of LLINs.

## Competing interests

The authors declare that they have no competing interests.

## Authors' contributions

EJK conceived the study, designed and coordinated the study, performed data analysis and draft the manuscript. SM and BJM participated in the field work and carried out the bioassays. MJM and HDM involved in statistical analysis. YEH drafted and reviewed the manuscript. MN reviewed the manuscript. AMM participated in coordinating bioassays and drafted part of the manuscript. All authors read and approved the final version of the manuscript.
